# Modelling Cumulative Effects of Air Pollution on Respiratory Illnesses by Performing Spline Estimation of Constrained, Additive Single-Index Model

**DOI:** 10.3390/toxics13030149

**Published:** 2025-02-21

**Authors:** Xingfa Zhang, Siyu Wang, Quanxi Shao, Sijia Wang, Yuezi Wei

**Affiliations:** 1 School of Economics and Statistics, Guangzhou University, Guangzhou 510006, China; xingfazhang@gzhu.edu.cn (X.Z.); wangsiyu202133@163.com (S.W.); 2 CSIRO Data61, P.O. Box 1130, Bentley, WA 6012, Australia; quanxi.shao@data61.csiro.au; 3 Ira A. Fulton Schools of Engineering, Arizona State University, 1151 S Forest Ave, Tempe, AZ 85281, USA; swang463@asu.edu; 4 School of Biomedical Engineering, Guangzhou Medical University, Guangzhou 511436, China

**Keywords:** air pollution, cumulative effect, respiratory illness, severe acute respiratory syndrome (SARS), spline estimation

## Abstract

It is widely recognised that air pollutants including sulphur dioxide (SO_2_), respirable suspended particulates (PM10), nitrogen oxides (NOx), nitrogen dioxide (NO_2_), and ozone (O_3_), as well as weather conditions such as temperature (Temp) and relative humidity (RH), are major causes of respiratory illnesses. To quantify the unknown and highly nonlinear relationships between these factors and respiratory illness, and the cumulative effect from exposure to symptoms, in this paper, we propose a semiparametric index model with constraints to capture the cumulative effect additively and the nonlinearity nonparametrically. As a case study, the model is applied to a dataset from the Hong Kong SAR. As the data period includes the SARS (severe acute respiratory syndrome) epidemic in 2003, we further construct a growth curve model to account for the extra impact of public health measures. The results show that the effects of SO_2_, NO_2_, and PM10 decay quickly, while the other pollutants have a period of stable accumulation (18–38 days for O_3_, 2–30 days for NOx, 1–13 days for RH, and 4–12 days for temperature). The results also show that the proposed model has a better fitting performance than previous models and hence has potential applications in health monitoring programs.

## 1. Introduction

Air pollution is among the major environmental problems in both developed and developing countries (WHO 2003) [1]. Many studies have addressed the impacts of air pollution on respiratory illnesses around the world (USEPA 1996) [2]. This research is motivated by the challenge of modelling the effect of ambient air pollution on respiratory illnesses. It is widely agreed that exposure to ambient air pollution may cause serious respiratory illnesses and that weather conditions may also contribute to their seriousness [3,4,5,6,7,8,9,10,11,12,13,14]. However, quantifying the effects of various pollutants as well as weather conditions is a difficult task due to the high nonlinearities in the impact of these environmental and weather factors on the onset of the respiratory illnesses and possible interactions amongst these factors. Unfortunately, the mechanism underlying such nonlinearities and interactions is unknown. To account for a possible incubative period and/or cumulative effects, we may consider the lags of potential factors that result in a large number of covariates in the regression. To address this issue, semiparametric models are frequently used, such as the cumulative effect model, index model, generalised additive model [15,16,17,18,19], and fuzzy process model [20,21]. The cumulative effect model has value in addressing the questions: “Is there a threshold below which no effects of the pollutants on health are expected to occur in all people?” and “What averaging period (time pattern) is the most relevant from the point of view of health?”, while the significant length of cumulative period has not been investigated.

The cumulative effects of pollutants and weather factors on respiratory illnesses have been recognised and investigated by researchers [22,23,24]. In data analyses, the ‘weekend-effect’ (change in hygiene habits) and environmental effects should be modelled simultaneously.

With a normal lifestyle, the effect of environmental and climatic factors on respiratory illness should be relatively stable, because the pattern of individual exposure to the environment and community is relatively stable. However, sudden events may change this established pattern and affect the number of cases of respiratory illnesses. This is true for the data we collected from Hong Kong, as the SARS (severe acute respiratory syndrome) epidemics in Hong Kong in 2003 changed people’s lifestyles during time [25]. There are at least two options for such a dataset. We can simply use the data within a period in which there was no significant event. Alternatively, we can introduce an extra component to model the effect of this significant event. In addition to allowing researchers a better understanding and use of the information, the latter approach can also provide insights into the impact of the significant event.

In this paper, we modify Xia and Tong’s (2006) cumulative model by including a component for the effect of SARS and determining the length of the cumulative period, to gain a guideline on the incubative period [9]. For computational efficiency, we also propose the use of the spline method for the effect function, which is more efficient than the local polynomial method. The paper is organised as follows. The data used in this study are described in the next section, followed by the modelling and estimation procedure. The results are then provided before a discussion and conclusions.

## 2. Dataset and Methods

### 2.1. Dataset

Hong Kong became a Special Administrative Region (HKSAR) of the People’s Republic of China on 1 July 1997, after a century and a half of British administration. It is located south of Mainland China with a population of 7 million and an area of 1103 km^2^ covering the Hong Kong Island, Kowloon, and the more rural New Territories. Its climate is sub-tropical, with temperatures between 10 degrees Celsius in winter and 33 degrees Celsius in summer.

Air pollution data were obtained from the Environmental Protection Department of HKSAR (www.epd.gov.hk) (accessed on 18 February 2025). These include the daily average levels of sulphur dioxide (SO_2_, μgm^−3^), respirable suspended particulates (PM10 μgm^−3^), nitrogen oxides (NOx, μgm^−3^), nitrogen dioxide (NO_2_, μgm^−3^), and ozone (O_3_, μgm^−3^). Weather data were obtained from the Hong Kong Observatory, including temperature (Temp, degrees Celsius) and relative humidity (RH). Instead of detailed concentrations of individual pollutants, the Air Pollution Index (API) is usually reported to the public. However, we did not use the API in this study as it constitutes secondary data derived from the highest indices of several key pollutants by comparing the measured concentrations with their respective health-related Air Quality Objectives (AQOs) established under the Air Pollution Control Ordinance.

**Figure 1:**
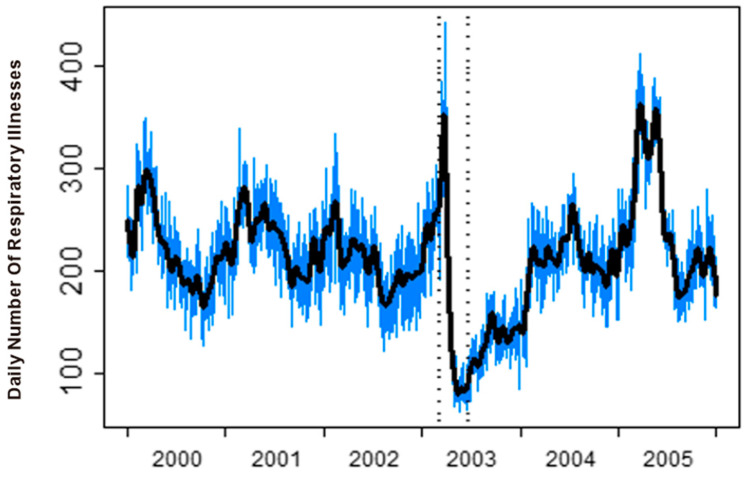
Time-series plot of the daily respiratory illness numbers. The thick black trend line is estimated by a smoothing spline. The two dotted vertical lines indicate the start and end of the SARS period. The source is Figure 2 of paper [25] (https://doi.org/10.1016/j.csda.2012.07.029) (accessed on 18 February 2025).

**Figure 2:**
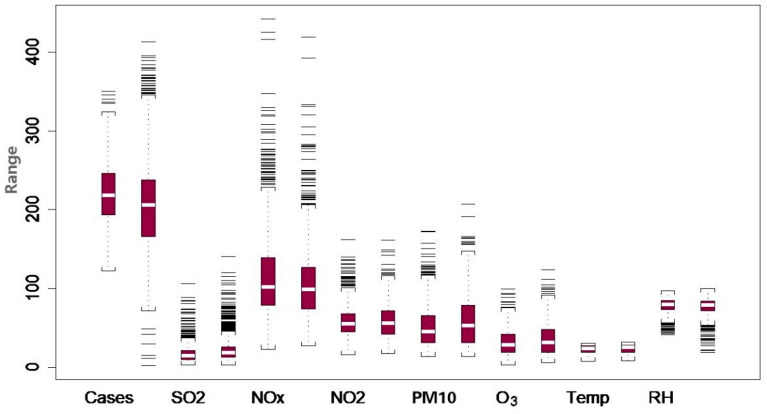
Box plot comparing daily values of variables before and after SARS epidemic. Note: For each item on the horizontal axis, the two box plots refer to values before and after SARS. The source is Figure 3 of paper [25] (https://doi.org/10.1016/j.csda.2012.07.029) (accessed on 18 February 2025).

Daily hospital admissions for respiratory diseases were obtained from the Hospital Authority of the HKSAR (the same data source used in Shao et al., 2010 and Wong et al., 2013 [10,25]). The period of study was from 1 January 2000 to 31 December 2005, totalling 2192 days. The number of respiratory illnesses is plotted in Figure 1. As we can see, there is a sudden drop following the outbreak of SARS. We divide the data into two parts. The pre-SARS part represents the data before 8 March 2003 and post-SARS after 23 June 2003 (inclusive). We do not use the data during the SARS epidemic, as this was a transition period of change. Visual comparisons in the form of box plots are given in Figure 2 for all variables. We can see that the temperature (with mean from 22.96 to 23.54, STD from 5.03 to 5.23) and RH (with mean from 78.01 to 76.92, STD from 9.72 to 11.21) are similar for the before- and after-SARS periods. However, SO_2_ (with mean from 16.95 to 22.60, STD from 11.14 to 16.43), PM10 (with mean from 51.04 to 58.07, STD from 24.89 to 31.32), and O_3_ (with mean from 31.80 to 35.45, STD from 15.70 to 19.89) increase, while NOx (with mean from 115.80 to 107.80, STD from 53.49 to 47.63) and NO_2_ (with mean from 57.63 to 58.23, STD from 19.32 to 22.11) are relatively stable. In contrast, the number of illnesses decreases (with mean from 220 to 207, STD from 38.91 to 64.11). For detailed summary statistics, refer to Table 1 in [25].

### 2.2. Methods

Let Y be our response variable, which is the number of daily admissions due to respiratory problems to a regional hospital, and $X=(X_{1},\;\cdots ,\;X_{p})$ be the covariates or variables affecting the response variable. The potential covariates in our study include sulphur dioxide (SO_2_), nitrogen dioxide (NO_2_), nitrogen oxides (NO_x_), respirable suspended particulates (PM10), ozone (O_3_), temperature (Temp), and relative humidity (RH). Let $t_{start}$ (=8 March 2003) and $t_{end}$ (23 June 2003) be the start and end of the SARS epidemic, respectively.

A general cumulative model is defined as

(1) $$Y_{t}=\mu +\lambda_{t}+S_{t}+g\left(X_{t}\right)+\varepsilon_{t},$$

where μ is a constant that appears in the model for model identification, representing the expectation of Y on the default day (Friday in our case) before SARS, with zero cumulative effects and the average humidity and temperature. Let

(2) $$\lambda_{t}=\left\{\begin{array}{cc} 0, & \mathrm{if}\;t\leq t_{start},\\ \xi_{1}+\frac{\xi_{3}}{1+\exp\left\{-\xi_{2}(t-\xi_{4})\right\}} & \mathrm{if}\;t\geq t_{end}, \end{array}\right.$$

model the impact of public health measures due to the SARS epidemic. Furthermore,

(3) $$S_{t}=\sum_{k=1}^{6}\alpha_{k}D_{k,t}$$

models the effect of the day of the week in the hospital admission system, with

(4) $$D_{k,t}=\left\{\begin{array}{cc} 1, & t\;\mathrm{is}\;\mathrm{the}\;k\mathrm{th}\;\mathrm{day}\;\mathrm{in}\;\mathrm{the}\;\mathrm{week},\\ 0 & \mathrm{Otherwise}, \end{array}\right.$$

Moreover,

(5) $$g(X_{t})=\sum_{j=1}^{p}g_{j}\left(\sum_{\tau =0}^{L_{j}}\theta_{j,\tau }X_{j,t-\tau }\right)$$

models the cumulative effect of environmental and climatic factors, where $X_{j,i}$ is the observation of the *j*th variable at time *i*, {$g_{j};j=1,\cdots ,p$ } are unknown functions, and $L_{j}$ is the length of the cumulative period of the *j*th variable (j=1,⋯,p). To ensure identifiability, we assume that $\sum_{\tau =0}^{L_{j}}\theta_{j,\tau }=1$ (j=1,⋯,p). For meaningful interpretation, it is also assumed that the effect functions for SO_2_, PM10, NO_x_, NO_2_, and O_3_ are monotonically non-decreasing and $\theta_{j,0}\geq \theta_{j,1}\geq \cdots \geq \theta_{j,L_{j}}\geq 0\;(j=1,\cdots ,p)$. The framework of (1) is similar to model (1) in Wong et al. (2013)’s paper [25], though the setting for unknown function $g(X_{t})$ is different. In [25], $g(X_{t})$ was set as a multi-index form with the purpose of dimension reduction, and it was difficult to directly study the cumulative effect of certain variables individually. In this paper, $g(X_{t})$ was set as an additive single-index form with constraints. Such a setting enables us to describe the cumulative effect functions for each considered variable.

Xia and Tong [9] recommended the backfitting algorithm together with the minimum average variance estimation (MAVE) and local polynomial method for the unknown functions {$g_{j};j=1,\cdots ,p$ }. However, the local polynomial approach is not computationally efficient for an index model with high dimensions because of the large matrix involved. Spline methods have a great computational advantage in approximating the unknown nonparametric effect function [26,27,28]. The number of knots and their locations need to be pre-determined in spline methods. Wang and Yang [28] suggested a set number of knots to be used based on the sample size and equally spaced knots for a uniform distribution. As the predictors in our model setting were not uniformly distributed, we determined the knot locations through the probability space of the empirical cumulative distribution, that is, the knots were equally spaced in the quantiles of the empirical distribution, giving roughly the same sample number in each segment.

For our additive single-index model, instead of updating all the cumulative effect functions simultaneously, we updated the functions separately by iteration. To do this, we needed an algorithm similar to the single-index model but with constraints. Following [28], the single-index model in our setting is estimated as below.

Let $Z_{t}$ be the residuals after removing all other intermediate effects during the iteration. To update the cumulative effect and the corresponding weights of a particular covariate variable, say the *j*th variable, we need to consider the estimation of a single-index model

(6) $$Z_{t}=g_{j}\left(\sum_{\tau =0}^{L_{j}}\theta_{j,\tau }X_{j,t-\tau }\right)+\varepsilon_{t}≜g_{j}(\theta_{j}^{T}X_{j,t})+\varepsilon_{t}$$

where $X_{j,t}=(X_{j,t},\;X_{j,t-1},\;\cdots ,\;X_{j,t-L_{j}}{)}^{T}$ (bear in mind that $X_{j,t}$ is a column vector) and $\theta_{j}=(\theta_{j,0},\;\theta_{j,1},\;\cdots ,\;\theta_{j,L_{j}}{)}^{T}$. Without constraints, the spline function can be formed as below [28].

For a fixed *θ_j_*, let

(7) $$U_{\theta_{j},t}=F_{L_{j}}(\theta_{j}^{T}X_{j,t}),\;(t=1,\;\cdots ,\;n)$$

where $F_{d}$ is the rescaled centred Beta{(d+1)/2,(d+1)/2)} cumulative distribution with

(8) $$F_{d}(v)=\int_{-1}^{\frac{v}{a}}\frac{\Gamma \left(d+1\right)}{\Gamma ([d+1]/2{)}^{2}2^{d}}(1-t^{2}{)}^{\frac{d-1}{2}}dt,\;v\in \left[-a,\;a\right].$$

In the implementation, *a* is chosen to be the 95th percentile of $\{||\;X_{j,t}||{\}}_{t=1}^{n}$, where

$$||X_{j,t}||=\max(|X_{j,t}|,\;|X_{j,t-1}|,\;\cdots ,\;|X_{j,t-L_{j}}|).$$

Under suitable assumptions, the regression function can be written in terms of $U_{\theta_{j},t}$ as

(9) $$\gamma_{\theta_{j}}(U_{\theta_{j},t})=g_{j}(\theta_{j}^{T}X_{j,t}),\;\mathrm{for}\;\mathrm{fixed}\;\theta_{j}.$$

To form the spline, we pre-select an integer $n^{1/6}\ll N=N_{n}\ll n^{1/5}{\left(\log n\right)}^{-2/5}$. Following [28], we use $N=\max\left\{n^{1/5.5},\;5\right\}.$ We divide [0, 1] into (*N* + 1) subintervals $J_{j}=[p_{j},\;p_{j+1})$ for j=0, 1, ⋯, N−1 and $J_{N}=[p_{N},\;1]$, where ${\left\{p_{j}\right\}}_{j=1}^{N}$ is a sequence of equally spaced points, called interior knots. We augment these interior points so that $p_{1-k}=\cdots =p_{-1}=p_{0}=0<p_{1}<\cdots <p_{N}<1=p_{N+1}=\cdots =p_{N+k}$. The *j*th B-spline of order *k* for this knot sequence, denoted by $B_{j,k}$, is recursively defined by [29].

For a fixed *θ_j_*, the cubic spline estimator ${\hat{\gamma }}_{\theta_{j}}$ of $\gamma_{\theta_{j}}$ and the corresponding estimator ${\hat{g}}_{j}$ of $g_{j}$ are

(10) $${\hat{\gamma }}_{\theta_{j}}(.)=\arg\underset{\gamma (.)\in \Gamma^{\left(2\right)}[0,1]}{min}\sum_{t=1}^{n}{\left\{Z_{t}-\gamma_{\theta_{j}}(U_{\theta_{j},t})\right\}}^{2},\;{\hat{g}}_{j}(v)={\hat{\gamma }}_{\theta_{j}}\{F_{L_{j}}(v)\}$$

where $\Gamma^{\left(2\right)}=\Gamma^{\left(2\right)}[0,\;1]$ is the space of all functions with the 2nd-order partial derivatives continuous on [0, 1] and which are polynomials of degree 3 on each interval. The estimator of the coefficient *θ_j_* is used to minimise the above objective function in the coefficient space. More detail can be found by referring to Section 3 of [28].

The monotonicity of *θ_j_* can be obtained by using a restricted optimisation method when minimising the objective function in Equation (10), and in our study, the Gradient Projection Method was adopted. The monotonicity of a certain effect function is guaranteed by monotonous B-spline coefficients that can be estimated simply by restricted linear regression. Bear in mind that, in our study, effect functions for SO_2_, PM10, NOx, NO_2_, and O_3_ were assumed to be non-decreasing. As the lag of the cumulative effect (i.e., the dimension of the single index) was unknown, a criterion was needed to determine the optimal lag. Information criteria are frequently used. Akaike’s information criterion [30] was adopted in this study due to its simplicity and powerfulness.

#### 2.2.1. Computing Algorithm with Given Lags

With given lags $L_{1},\cdots ,L_{p}$ for the cumulative effect, model (1) can be estimated by the following steps. Before the start of the iteration, the initial estimates of all parameters and functions are set by the following procedure:

Step 1. Initialise the estimates of constant μ and the parameter in the weekly effect S(t) and SARS effect λ(t) by least-squares fitting of $Y_{t}$ on S(t) and λ(t) with a constant term. Denote the estimates as ${\hat{\mu }}^{\left[0\right]}$,${\hat{\xi }}^{\left[0\right]}$ and ${\hat{\phi }}^{\left[0\right]}$.

Step 2. Let $e_{t}^{\left[0\right]}=Y_{t}-{\hat{\mu }}^{\left[0\right]}-{\hat{S}}^{\left[0\right]}(t)-{\hat{\lambda }}^{\left[0\right]}(t)$, where ${\hat{S}}^{\left[0\right]}(t)$ and ${\hat{\lambda }}^{\left[0\right]}(t)$ are the fitted values using ${\hat{\xi }}^{\left[0\right]}$ and ${\hat{\phi }}^{\left[0\right]}$, respectively.

Step 3. The cumulative effect functions and the corresponding weights are initialised as below.

Step 3.1. Estimate the *first* cumulative effect function and the corresponding weights using the single-index model (6) with $Z_{t}=e_{t}^{\left[0\right]}$ and $U_{t}=X_{1,t}$. Denote the estimates of the cumulative effect function and the corresponding weights by ${\hat{g}}_{1}^{\left[0\right]}(.)$ and ${\hat{\theta }}_{1}^{\left[0\right]}=({\hat{\theta }}_{1,0}^{\left[0\right]}\cdots ,\;{\hat{\theta }}_{1,L_{1}}^{\left[0\right]}{)}^{T}$, respectively.

Step 3.2. For j=2, ⋯, p, estimate the *j*th cumulative effect function and the corresponding weights using the single-index model (6) with $Z_{t}=e_{t}^{\left[0\right]}-\sum_{i=1}^{j-1}{\hat{g}}_{i}^{\left[0\right]}\left(\sum_{\tau =0}^{L_{i}}{\hat{\theta }}_{i,\tau }^{\left[0\right]}X_{i,t-\tau }\right)$ and $U_{t}=X_{j,t}$. Denote the estimates of the cumulative effect function and the corresponding weights by ${\hat{g}}_{j}^{\left[0\right]}\left(.\right)$ and ${\hat{\theta }}_{j}^{\left[0\right]}=({\hat{\theta }}_{j,0}^{\left[0\right]}\cdots ,\;{\hat{\theta }}_{j,L_{j}}^{\left[0\right]}{)}^{T}$, respectively.

{End}

Once the initialisation is performed, the iteration can be implemented as below.

Step 4. In the *m*th iteration, (*m* = 1, 2, …), compute and update the constant μ and the coefficients of the weekly effect and SARS effect denoted by ${\hat{\mu }}^{\left[m\right]}$, ${\hat{\xi }}^{\left[m\right]},$ and ${\hat{\phi }}^{\left[m\right]}$ using least-squares fitting of $Y_{t}^{\left[m\right]}$ on S(t) and λ(t) with a constant term.

$$Y_{t}^{\left[m\right]}=Y_{t}-\sum_{j=1}^{p}{\hat{g}}_{j}^{\left[m-1\right]}\left(\sum_{\tau =0}^{L_{j}}{\hat{\theta }}_{j,\tau }^{\left[m-1\right]}X_{j,t-\tau }\right),$$

Step 5. Let $e_{t}^{\left[m\right]}=Y_{t}-{\hat{\mu }}^{\left[m\right]}-{\hat{S}}^{\left[m\right]}(t)-{\hat{\lambda }}^{\left[m\right]}(t)$, where ${\hat{S}}^{\left[m\right]}(t)$ and ${\hat{\lambda }}^{\left[m\right]}(t)$ are the fitted values using ${\hat{\xi }}^{\left[m\right]}$ and ${\hat{\phi }}^{\left[m\right]}$, respectively.

Step 6. The cumulative effect functions and the corresponding weights are estimated as below.

Step 6.1. Estimate the *first* cumulative effect function and the corresponding weights using the single-index model (6) with $Z_{t}=e_{t}^{\left[m\right]}$ and $U_{t}=X_{1,t}$. Denote the estimates of the cumulative effect function and the corresponding weights by ${\hat{g}}_{1}^{\left[m\right]}(.)$ and ${\hat{\theta }}_{1}^{\left[m\right]}=({\hat{\theta }}_{1,0}^{\left[m\right]}\cdots ,\;{\hat{\theta }}_{1,L_{1}}^{\left[m\right]}{)}^{T}$, respectively.

Step 6.2. For j=2, ⋯, p, estimate the *j*th cumulative effect function and the corresponding weights using the single-index model (6) with $Z_{t}=e_{t}^{\left[m\right]}-\sum_{i=1}^{j-1}{\hat{g}}_{i}^{\left[m\right]}\left(\sum_{\tau =0}^{L_{i}}{\hat{\theta }}_{i,\tau }^{\left[m\right]}X_{i,t-\tau }\right)$ and $U_{t}=X_{j,t}$. Denote the estimates of the cumulative effect function and the corresponding weights by ${\hat{g}}_{j}^{\left[m\right]}\left(.\right)$ and ${\hat{\theta }}_{j}^{\left[m\right]}=({\hat{\theta }}_{j,0}^{\left[m\right]}\cdots ,\;{\hat{\theta }}_{j,L_{j}}^{\left[m\right]}{)}^{T},$ respectively.

Step 6.3. Update the residual by $e_{t}^{\left[m\right]}\leftarrow e_{t}^{\left[m\right]}-\sum_{i=1}^{p}{\hat{g}}_{i}^{\left[m\right]}\left(\sum_{\tau =0}^{L_{i}}{\hat{\theta }}_{i,\tau }^{\left[m\right]}X_{i,t-\tau }\right)$ and check the convergence. If the convergence criterion is not met, go to Step 4.

{End}

#### 2.2.2. Search Approach for (Locally) Optimal Lags

In Steps 1–6 in the above algorithm, for given lags $L_{1},\cdots ,L_{p}$, one can estimate model (1) and calculate the related AIC (Akaike information criterion) value, denoted as AIC ($L_{1},\cdots ,L_{p}$). The AIC is a measure of the goodness of fit of a statistical model. Assuming that the model errors are independently normally distributed, k is the number of parameters in the fitted model, n is the number of observations, and SSR is the sum of squared residuals; then, AIC becomes

AIC = 2k + nln(SSR/n).

Due to the fixed sample size, AIC ($L_{1},\cdots ,L_{p}$) can be calculated by

$$\mathrm{AIC}\;(L_{1},\cdots ,L_{p})=\log\left(MSE\right)+2m/n,$$

where MSE is the mean squared error, and m is number of parameters, which includes both spline parameters and model parameters (however, the spline parameters are not shown in the arguments of the AIC definition for ease of notation and can be ignored in the AIC calculation because their numbers are fixed and therefore do not affect the order of AIC values). Next, we give a method to estimate the (locally) optimal lags for model (1) based on AIC values.

Step 1. Set $L_{1}=\cdots =L_{p}=k$, with k taking values from the integer set: $\left\{1,1+l,1+2l,\cdots ,K\right\}$. Here, l is a gap between integers and K is a given upper bound for lag estimation. Denote the minimum point of the AIC ($L_{1}=k,\cdots ,L_{p}=k$) with respect to k to be $k^{\left[0\right]},$ and then the initial estimates for lags are set as $L_{1}^{\left[0\right]}=\cdots =L_{p}^{\left[0\right]}=k^{\left[0\right]}$.

Step 2. To renew the values of lag $L_{j},j=1,\cdots ,p,$ let $L_{i}=L_{i}^{\left[0\right]},(i\neq j,i=1,\cdots ,p)$, $L_{j}=k$, with k taking integer values from the interval $\left[L_{j}^{\left[0\right]}-d,L_{j}^{\left[0\right]}+d\right]$. Here, d is a relatively small integer. Find the minimum point of

$$\mathrm{AIC}\;(L_{1}=L_{1}^{\left[0\right]},\cdots ,L_{j-1}=L_{j-1}^{\left[0\right]},L_{j}=k,L_{j+1}=L_{j+1}^{\left[0\right]},\cdots ,L_{p}=L_{p}^{\left[0\right]})$$

with respect to k and denote it as $k_{j}^{\left[1\right]}$. Then, the updated value for $L_{j}$ is $L_{j}^{\left[1\right]}=k_{j}^{\left[1\right]}$.

Step 3. Check the convergence by comparing the distance between ($L_{1}^{\left[0\right]},\cdots ,L_{p}^{\left[0\right]}$) and ($L_{1}^{\left[1\right]},\cdots ,L_{p}^{\left[1\right]}$). If the convergence criterion is not met, set $L_{j}^{\left[0\right]}=L_{j}^{\left[1\right]}(j=1,\cdots ,p)$ and go to Step 2.

In our study, the above Step 1 was executed with l=10,K=200 and d in Step 2 was set to be 5.

{End}

Finally, we used the bootstrap method to calculate confidence bounds for the estimates.

## 3. Results and Discussion

To obtain an approximate normal distribution, we applied the logarithmic transform to the number of daily respiratory illnesses as usual. Unlike the previous studies, which used fixed lags of the cumulative effect, this study estimated the relatively optimal lags for each predictor. The weekly effect due to hospital admission and the SARS effect due to habit change were considered as before. The maximum lag was pre-determined as 200 days. Friday was used as the default day in the weekly effect component (Equation (3)).

**Figure 3:**
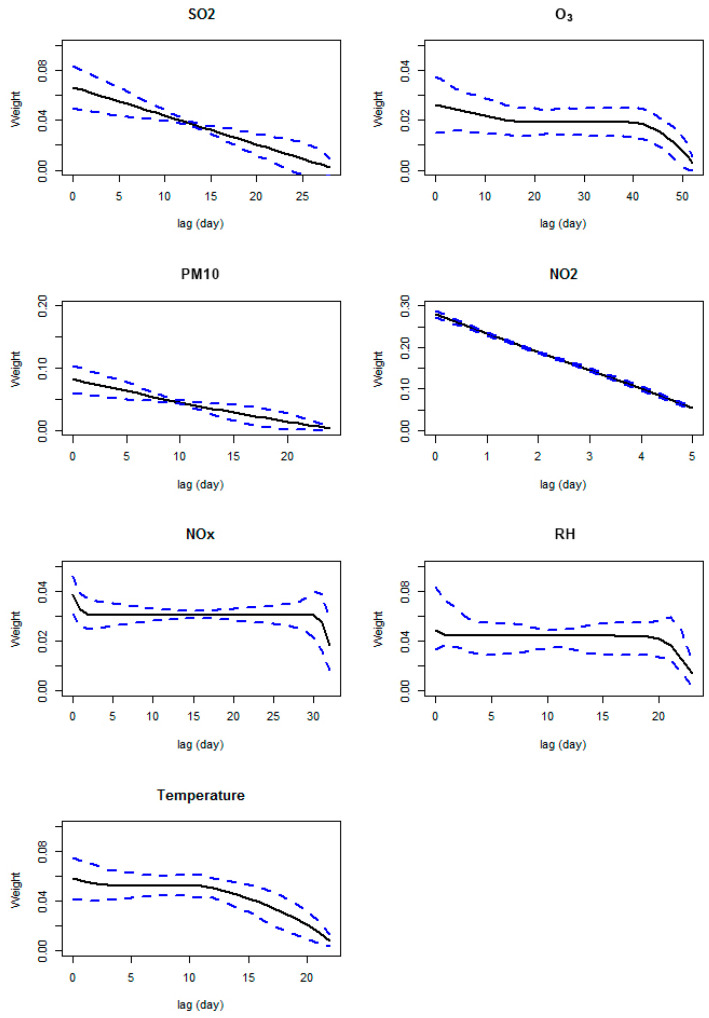
Weight plots (black lines), together with corresponding 95% confidence intervals (blue dashed lines), against the lags for individual predictors.

**Figure 4:**
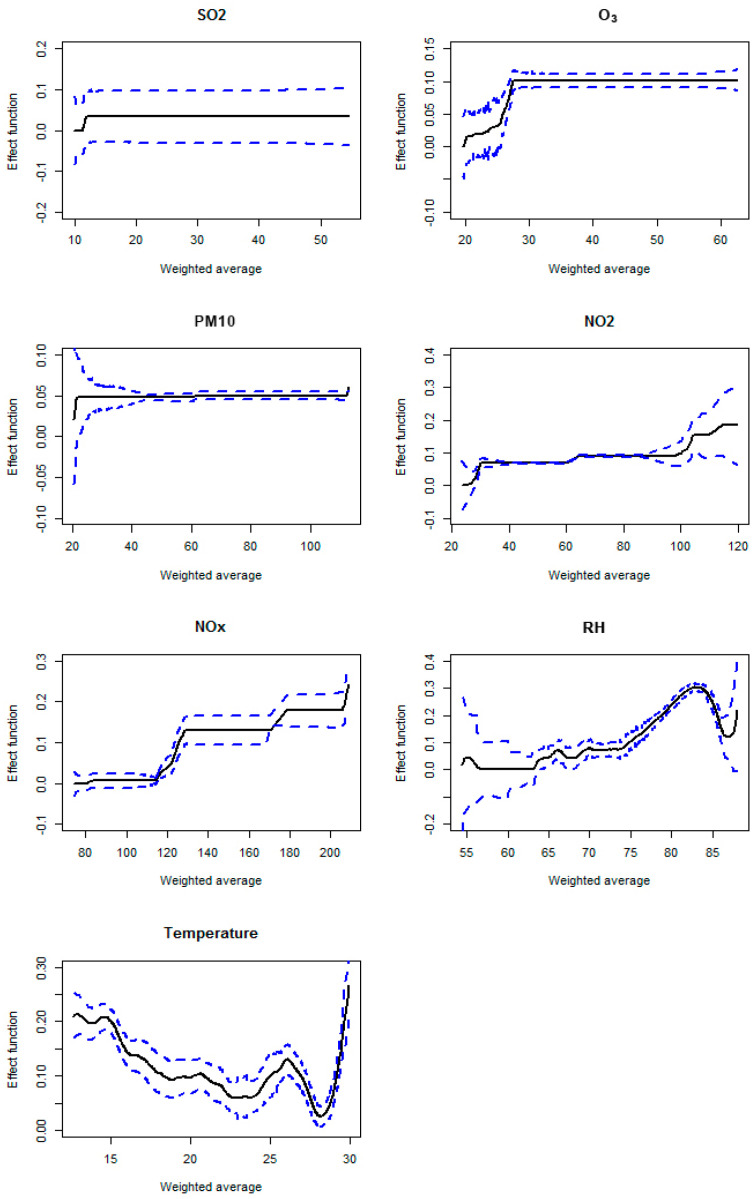
Cumulative effect functions plots (black lines), together with corresponding 95% confidence intervals (blue dashed lines), against the weighted averages for individual predictors.

The optimal lags affecting respiratory illnesses are given in Table 1, and the associated weights are depicted in Figure 3. It can be seen that O_3_ has the longest cumulative effect on the respiratory illnesses and NO_2_ has the shortest cumulative effect, while the other pollutants have similar durations. The effects by SO_2_, NO_2_, and PM10 decay quickly, while the other pollutants have a period of accumulation (18–38 days for O_3_, 2–30 days for NOx, 1–13 days for RH, and 4–12 days for temperature). The cumulative effects of individual predictors are plotted in Figure 4 against the weighted averages. It can be seen that the effects of pollutants tend to level up after certain cumulative averages. Such results are consistent with those of [9], but the threshold point and the effect function range for each pollutant are different. This could be due to the fact that the model covariates and constraints are different. While the cumulative effects of the pollutants are comparable to those of [9], the cumulative effects of weather factors (RH and temperature) are different from the previous findings and are of interest. It can be seen that the RH has the least effect on respiratory illnesses at around 60%, which is the most comfortable level for human beings, and reaches the highest effect at around 80%. This finding is consistent with [31]. For temperature, both the cold and hot weather tend to increase the incidence of respiratory diseases, and the most suitable temperature is around 28 degrees Celsius. However, there is a local peak at around 26 degrees Celsius, which may relate to the later spring and early autumn, during which the temperatures are not settled and, as a result, may cause slightly more incidents.

**Figure 5:**
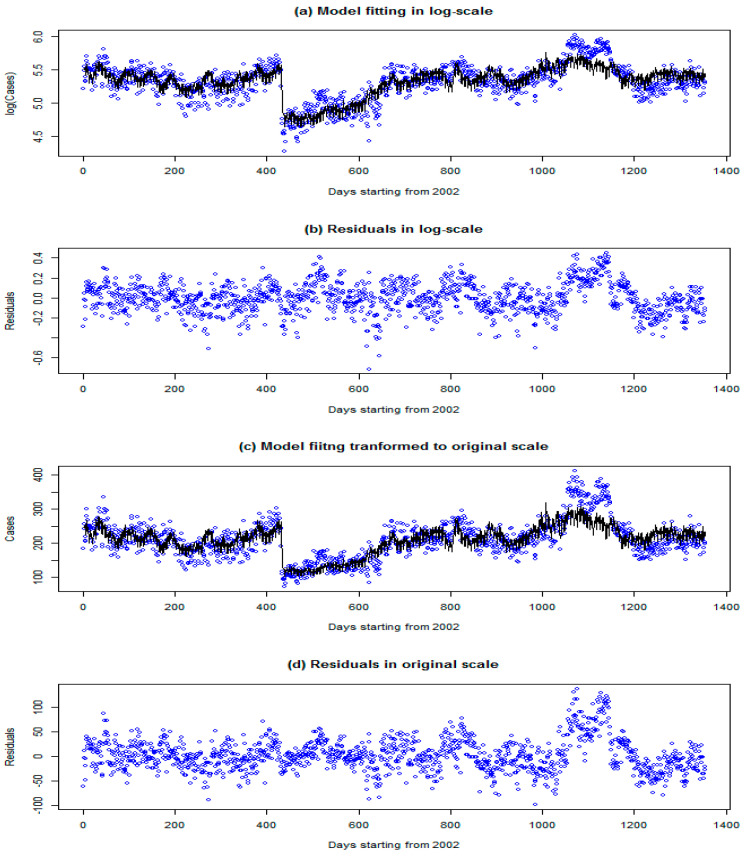
Time-series plots of fitting values (black lines) together with the observations (blue dots) in the log scale (**a**) and transformed to the original scale (**c**). The residuals are also plotted in the log scale (**b**) and the original scale (**d**), directly calculated as the difference between the original observation and the exponential of the fitted values.

As by-products, the parameter estimates for the constant term μ (Equation (1)), weekly effect (Equation (3)), and health measures due to the SARS epidemic (Equation (5)) are given in Table 2. The results are comparable with the results in [25]. The number of reported cases on the weekend is less than that on weekdays. The health measures imposed during the SARS epidemic had a significant effect on the reported cases. The model fitting with the observations and the residuals is given in Figure 5, from which it can be seen that the fitted values capture the observation trend well, with the residuals approximating a white noise process. The root mean squared error between observed and fitted values is 0.1515 (versus 0.1802 for the model in [25]), which translates to RMSE = 32.50 for the original number of cases (versus 37.25 for the model in [25]). This implies that our model’s fitting performance is slightly better than those of previous models.

## 4. Summary and Conclusions

This paper proposes an estimation method based on splines for a constrained, additive single-index model, motivated by modelling the effect of air pollution on respiratory illnesses. The additivity is based on the consideration of multiple covariates, and the single index is for the cumulative effect. The monotonic constraint is used to model the decreasing effect over time. The spline method is used in the non-parametric effect functions due to its computing efficiency. The bootstrap is used to evaluate the uncertainty bounds in the model parameters. An algorithm was designed and implemented in MATLAB 2023.

When modelling the effect of air pollution on respiratory illnesses in Hong Kong during 2000 to 2005, the weekly effect and impact of health measures during the SARS epidemic were modelled by day-of-the-week variables and the growth curve function, respectively. The results showed that the pollution variables had similar cumulative periods of around one month, except NO_2_ with the shortest period (5 days) and O_3_ with the longest period (52 days). These results may aid further theoretical and practical studies of air pollution. The effects of weather factors are consistent with the existing findings and can be explained by common knowledge in public health. The weekly effect and impact of the SARS epidemic are consistent with previous studies, and the fitting of the model is better. Consequently, the model framework and algorithm can be used for other applications where a similar model structure is applicable.

Related issues also need to be further studied, including the asymptotic properties of model estimators and variable selection. Then, confidence intervals based on the asymptotic variance may be obtained, which could be more accurate than those obtained by the bootstrap method. It also makes sense to try different variables or consider the interactive effect between variables in explaining respiratory illnesses. For example, jointly considering NO with NO2 could make more sense than the case of NOx with NO2, because NOx is the sum of NO2 and NO. We leave these considerations for future research.

## Figures and Tables

**Table 1:** Optimal lags of individual pollutants, humidity, and temperature.

Predictor	SO_2_	O_3_	PM10	NO_2_	NOx	RH	Temp.
lag	28	52	24	5	32	23	22

**Table 2:** Parameter estimates, and their standard deviations and 95% confidence bounds.

Parameter	μ	$\xi_{1}$	$\xi_{2}$	$\xi_{3}$	$\xi_{4}$	$\alpha_{1}$	$\alpha_{2}$	$\alpha_{3}$	$\alpha_{4}$	$\alpha_{5}$	$\alpha_{6}$
Estimate	4.610	−0.588	0.010	0.747	203.36	0.070	0.077	0.237	0.155	0.170	0.191
Std.	0.114	0.067	0.002	0.073	22.24	0.033	0.031	0.035	0.032	0.036	0.036
2.5% bound	4.385	−0.720	0.006	0.605	159.78	0.005	0.016	0.169	0.092	0.099	0.121
97.5% bound	4.834	−0.456	0.014	0.890	246.94	0.135	0.139	0.305	0.219	0.241	0.262

## Data Availability

The data will be available on request from the corresponding author.
